# Spatial and Temporal Variations of Heavy Metals’ Bioavailability in Soils Regulated by a Combined Material of Calcium Sulfate and Ferric Oxide

**DOI:** 10.3390/toxics11040296

**Published:** 2023-03-24

**Authors:** Chi Zhang, Jie Li, Yuxia Dai, Williamson Gustave, Weiwei Zhai, Zhong Zhong, Jianmeng Chen

**Affiliations:** 1 Key Laboratory of Microbial Technology for Industrial Pollution Control of Zhejiang Province, College of Environment, Zhejiang University of Technology, Hangzhou 310058, China; 2 Zhejiang Key Laborary of Environmental Protect Technology, Eco-Environmental Sciences Research & Design Institute of Zhejiang Province, Hangzhou 310007, China; 3 Zhejiang Provincial Key Laboratory of Agricultural Resources and Environment, Institute of Soil and Water Resources and Environmental Science, College of Environmental and Resource Sciences, Zhejiang University, Hangzhou 310058, China; 4 School of Chemistry, Environmental & Life Sciences, University of the Bahamas, New Providence, Nassau P.O. Box N-4912, Bahamas

**Keywords:** soil, heavy metals, in-situ immobilization, bioavailability, spatial and temporal variations

## Abstract

Heavy metal pollution in soils threatens food safety and human health. Calcium sulfate and ferric oxide are commonly used to immobilize heavy metals in soils. However, the spatial and temporal variations of the heavy metals’ bioavailability in soils regulated by a combined material of calcium sulfate and ferric oxide (CSF) remain unclear. In this work, two soil column experiments were conducted to investigate the spatial and temporal variations of CSF immobilized Cd, Pb, and As. In the horizontal soil column, the results showed that CSF’s immobilization range for Cd increased over time, and adding CSF in the center of the soil column decreased the concentrations of bioavailable Cd significantly, up to 8 cm away by day 100. The CSF immobilization effect on Pb and As only existed in the center of the soil column. The CSF’s immobilization depths for Cd and Pb in the vertical soil column increased over time and extended to 20 cm deep by day 100. However, the CSF’s immobilization depths for As only extended to between 5 and 10 cm deep after 100 days of incubation. Overall, the results from this study can serve as a guide to determine the CSF application frequency and spacing distance for the in-situ immobilization of heavy metals in soils.

## 1. Introduction

Soil heavy metal pollution has increased significantly over the past 50 years due to increased anthropogenic activities associated with rapid urbanization and industrialization. This increase in soil contaminants poses a serious risk to cultivated land quality and food safety globally [1,2,3]. In China, soil heavy metal pollution is a serious threat and has attracted considerable attention. According to the National Soil Pollution Survey Bulletin issued by the Ministry of Environmental Protection (MEP) and the Ministry of Land and Resources in 2014, 16% of all the arable soils in China exceeded the national standard for soil pollutants (GB15618-1995). Among them, the excessive rates of cadmium (Cd), mercury (Hg), arsenic (As), lead (Pb), and chromium (Cr) were 7.0%, 1.6%, 2.7%, 1.5%, and 1.1%, respectively. Yang et al. conducted an integrated analysis of the concentration of heavy metals in the soil of 1041 agricultural lands in China. They found that heavy metals such as Cd, Pb, and As and their related hazards were particularly serious [2]. These heavy metals in soil can enter the human body through oral and nasal inhalation, dermal contact, and the food chain, ultimately affecting human health [4]. Therefore, the protection and remediation of heavy metal-contaminated soil are essential.

Unlike organic pollutants, heavy metals are non-degradable and persistent in the soil environment. Therefore, once heavy metals contaminate the soil, the contaminants’ concentration and ecological toxicity will persist until they have transformed into a less toxic form or are completely removed [5]. Various remediation technologies have been developed, including physical, chemical, and biological remediation techniques, to decrease soil heavy metals’ bioavailability and toxicity [6]. For example, phytoremediation is considered an efficient approach to reducing the concentration of heavy metals in soil [7,8]. The replacement technique is used to remediate contaminated soil by completely or partially replacing contaminated soil with clean soil [9]. Among these techniques, in-situ immobilization is a promising soil remediation technology that employs stabilizers to decrease heavy metals’ bioavailability. In-situ immobilization has the advantages of practicability, rapid results, and cost-effectiveness [10]. The stabilizer selection is the most crucial criterion for the success of in-situ immobilization. The common stabilizers used for heavy metal immobilization include organic stabilizers, inorganic stabilizers, and organic-inorganic composite stabilizers, such as lime, fly ash, and biochar [11,12]. As cost-effective and environmentally friendly stabilizers, more and more iron-based materials are used for the immobilization of heavy metals, such as zero-valent iron, oxides, iron sulfides, and loaded iron-based materials [13,14], which have a high specific surface area, strong redox capacity, and expand the range of the effective working pH. Yang et al. found that biochar-supported nanoscale zero-valent iron could transform the fraction of unstable heavy metals into a stable form, which substantially decreased the availability of the heavy metals and hence greatly reduced the human health exposure risk [15]. Two iron-based materials, 2-line ferrihydrite and goethite, promote Cd transformation to more stable speciation in contaminated soil [16]. Our previous studies have shown that a combined material of calcium sulfate and ferric oxide (CSF) can effectively decrease the mobility and bioavailability of Cd, Pb, and As in paddy soils [17,18].

The stabilization efficiency was affected by heavy metals and the dosage of stabilizers. For example, Wang et al. showed that 1% and 0.5% biochar had significantly different impacts on Cd in rice roots [12]. The lime, fly ash, and biochar could increase soil pH and decrease Zn, Cd, Cu, and Pb concentrations while increasing the As concentrations [19]. In addition, the immobilization efficiency of heavy metal-contaminated soil is influenced by the stability time. Cui et al. found that adding soil amendments such as apatite and charcoal to contaminated soil can effectively reduce the leaching rates and bioavailability of Cu and Cd in the soil, but both will gradually increase with time [20]. However, there are few studies on the pollution repair process of heavy metal-contaminated soils by composite materials and their stability, timeliness, and effective diffusion range. Due to the convenience of soil column experience, previous scientists usually used soil column experiments to verify heavy metals’ temporal and spatial migration characteristics [21,22,23,24]. Therefore, CSF as an effective immobilization and remediation material in soils contaminated by Cd, Pb, and As, however, the adequate time and remediation range of CSF needs to be further studied to determine the ideal application frequency and CSF concentration required to reduce the cost and prevent soil hardening.

In this study, two soil column experiments were set up to (1) evaluate the efficiency and persistence of CSF for decreasing the bioavailability of Cd, Pb, and As in paddy soil; and (2) explore the spatial and temporal variation of the bioavailability of Cd, Pb, and As regulated by CSF. Our study aimed to provide new insights for rational and effective remediation procedures for heavy metal-contaminated soils by CSF.

## 2. Materials and Methods

### 2.1. Soil and CSF Characterization

Soil samples were collected from the surface layer (that is, from 0 to 20 cm) of a contaminated paddy field near a mining area located in Shangyu, Zhejiang, China (120°87′ E, 30°03′ N). The sampling site has a typical subtropical climate. The basic physical and chemical properties of soil are given in Table 1. The soil pH was 6.48, and the organic matter was 43.33 g kg^−1^. The Cd, Pb, and As concentrations in the soil were 0.54 mg kg^−1^, 416.58 mg kg^−1^, and 94.20 mg kg^−1^, respectively. The CSF was prepared by mixing calcium sulfate (CaSO_4_·2H_2_O) and ferric oxide (Fe_2_O_3_) at a ratio of 9:1. The CaSO_4_**·**2H_2_O and Fe_2_O_3_ were purchased from Zibo Jinshun Chemical Industry Co., Ltd. (Zibo, China) and Sinopharm Chemical Reagent Co., Ltd. (Shanghai, China), respectively. The CSF was ground and passed through a 0.15 mm nylon mesh. The mean particle size was 10.37 µm, and the specific surface area of CSF was 0.65 m^2^ g^−1^. The characteristics of the CSF stabilizer can be found in Table 1.

### 2.2. Experimental Design

**Figure 1:**
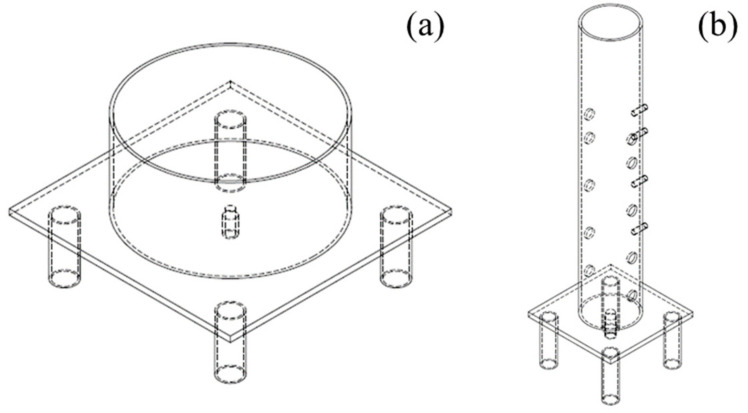
Schematic drawing of the soil columns used in the experiments. (**a**): Horizontal soil column; (**b**): Vertical soil column.

Two different soil column experiments were conducted to explore the spatial variation of Cd, Pb, and As bioavailability regulated by CSF. One was a horizontal soil column, which was used to examine the effect of CSF on the bioavailability and migration of Cd, Pb, and As in the horizontal space (Figure 1a). The main part of the horizontal soil column was 15 cm high and 30 cm in diameter. The other was a vertical soil column, which was used to explore the effect of CSF on the bioavailability and migration of Cd, Pb, and As in the vertical space (Figure 1b). The main part of the vertical soil column was 61 cm high and 10 cm in diameter. A layer of nylon gauze was placed at the bottom of the soil column container, followed by a layer of 3 and 5-cm thick quartz sand in horizontal and vertical soil column containers, respectively. After that, the soil was used to fill in the layers 5 cm apart. The soil was weighed before use to ensure the soil bulk density was the same in each layer. Each soil layer was packed with tamping, especially around the side wall of the soil column container, to prevent the side wall from collapsing. A 10 cm diameter cylindrical gauze bag was loaded in the center for the horizontal soil column experiment. The CSF treatment of the horizontal soil column (CSF-H) was loaded with the original soil mixed with 0.15% CSF in the gauze bag. The control of the horizontal soil column (CK-H) was loaded with the original soil in the gauze bag without CSF. A layer of gauze was laid on the soil layer 38 cm from the bottom for the vertical soil column experiment. The original soil 15 cm thick was used as the control treatment in the vertical soil column (CK-V), and the original soil was mixed with 0.15% CSF of 15 cm thick as CSF in the treatment of the vertical soil column (CSF-V).

The soil column experiments were placed indoors at 25 °C, and three replicates were set for each treatment. At the beginning of the experiment, deionized water was added to saturate the soil, and then the natural environment was simulated to alternate between dry and wet soil cycles. Based on rainfall at the sampling site and the local fields’ water management strategies, we added water to the soil column every three days, with 2000 mL water each time for the horizontal soil column and 250 mL water for the vertical soil column. Soil porewater samples were taken 15, 30, 60, and 100 days after the saturation of the soil column using a Rhizon sampler (2.5 cm × 10 cm, MOM, Rhizon, The Netherlands). The soil porewater samples of the horizontal soil column were taken from the gauze bag in the central soil column (0 cm) and 1, 4, and 8 cm away from the central soil column. The soil porewater samples of the vertical soil column were collected at 0, 5, 10, 20, 30, and 38 cm from the top of the soil column. The collected soil porewater was acidified with 6 M hydrochloric acid to prevent heavy metal precipitation and transformation [25].

### 2.3. Chemical Analysis of Soil

Soil pH was determined in a 1:2.5 ratio soil/water suspension. The S (SO_4_^2−^) was determined by the barium sulfate turbidimetric method. The bioavailable Cd, Pb, As, and total Fe in soil porewater were determined by inductively coupled plasma mass spectrometry (ICP-MS NEXION300X, PerkinElmer, Inc., Shelton, CT, USA). See the previous study for details [17]. The standard internal method was used to determine the accuracy of the analytical methods. A standard sample of 50 µg L^−1^ was measured after every ten samples as a quality control measure. The recoveries of internal standards for bioavailable heavy metal were within the range of 95.2% and 106.3%, which proved that the detection method was credible.

### 2.4. Data Analysis

SPSS 19.0 (SPSS, Chicago, IL, USA) software was used for the statistical analysis. The one-way ANOVA followed by the least significant difference (LSD) was used on normally distributed data, and not normally distributed data were compared by the Kruskal–Wallis test. A *p*-value of less than 0.05 was considered statistically significant. The data in the figures and tables show the average ± standard deviation.

## 3. Results and Discussion

### 3.1. Effect of CSF on pH

In the horizontal soil column, the soil column center’s pH decreased significantly by 12.2% and 10.2% on days 15 and 100 with the addition of CSF (Table 2). However, the soil pH at different distances from the central soil column (1 cm, 4 cm, and 8 cm) showed no significant change in the CK-H and CSF-H treatments. The soil pH on day 100 was slightly higher than on day 15, which was inconsistent with previous observations [1,26]. This may have occurred because the main components of CSF are CaSO_4_·2H_2_O and Fe_2_O_3_. Previous studies have shown that the decrease in soil pH with CSF addition is due to increased sulfate (SO_4_^2−^) concentrations [27]. In addition, the soil was not flooded during the incubation, possibly hindering sulfate reduction and thereby limiting proton consumption in the soil.

In the vertical soil column, the soil layer of 5 to 38 cm from the topsoil’s pH was higher than that of the topsoil on days 15 and 100 in both the CK and CSF treatments (Table 3). Moreover, on the 100th day after adding the CSF, the soil pH 5 cm away from the topsoil was significantly lower than that of the topsoil. The soil pH decreased by 6.3% in treatment 5 cm away from the topsoil. The pH of the CSF treatments showed an increasing trend with the soil depth. It could be that simulated rainfall washes some acid-causing ions into the lower layers, which acidifies the soil.

### 3.2. Effect of CSF on the Concentrations of SO_4_^2−^ and Total Fe

The SO_4_^2−^ concentrations of the soil column center in the horizontal soil column were significantly higher than that in the soil 1 to 8 cm away from the soil column center (Table 4) in the CSF-H treatment. The SO_4_^2−^ concentrations decreased with increasing distance from the soil column center. On day 15, after adding CSF, the SO_4_^2−^ concentrations in the center soil and 1 cm away from the soil column center significantly increased by 628.1% and 326.3%, respectively. On day 100, after adding CSF, the SO_4_^2−^ concentration in the center of the soil column and the soil 1 cm, 4 cm, and 8 cm away from the soil column center significantly increased by 286.8%, 236.0%, 55.3%, and 86.3%, respectively, compared with the CK treatment. The increase in SO_4_^2−^ concentrations may be ascribed to the added CaSO_4_ through the CSF. Inorganic SO_4_^2−^ in the soil solution is highly mobile [28].

On day 15, after adding CSF, the total Fe content in the center of the soil column was significantly higher than that in the soil 1 to 4 cm away from the soil column center (Table 4). The total Fe concentrations in the center of the soil column with the addition of CSF significantly increased by 102.7% compared with the CK treatment on day 15, while the total Fe concentrations in the soil 1 to 8 cm away from the center of the soil column were not significantly affected. The total Fe concentrations in the soil 1 cm away from the central soil column with CSF addition were significantly increased by 158.1% after 100 days. However, the CSF addition had no significant influence on the Fe contents in the other soils. In the CK and CSF treatments, the total Fe concentrations decreased on day 100 compared with day 15 at the same distance. The decrease after 100 days may be because by then, Fe oxides and Fe hydroxides in the soil were being reduced and started to dissolve [29,30], thereby increasing the Fe contents in the deeper layers.

In the vertical soil column, the concentrations of SO_4_^2−^ in the CK treatment showed no significant changes between the distances from the topsoil on day 15. At the same time, the concentration of SO_4_^2−^ in the topsoil significantly increased by 74.4% with the addition of CSF compared with the CK. Moreover, the concentrations of SO_4_^2−^ at 5, 10, and 20 cm away from the topsoil significantly decreased by 68.8%, 58.8%, and 45.5%, respectively (Table 5), in the CSF treatment. Compared with the concentration of SO_4_^2−^ in the topsoil layer, the SO_4_^2−^ concentrations in the soil 5 cm and 10 cm away from the topsoil layer significantly decreased by 65.7% and 45.3%, respectively, in the CK treatment after 100 days. In the CSF treatment, the concentration of SO_4_^2−^ in the soil layer from 5, 10, 20, and 30 cm away significantly decreased by 48.5%, 69.0%, 61.5%, and 46.5%, respectively. Previous studies have reported that mineralogical composition, total carbon, particle-size distribution, pH, and the presence of other ions could influence the adsorbed SO_4_^2−^ in the soil [31,32]. The concentration of SO_4_^2−^ in the soil layer from 5, 10, 20, and 30 cm away significantly decreased, indicating that SO_4_^2−^ had been reduced. In addition, SO_4_^2−^ could be retained by colloidal Fe oxides or complexed by Fe oxides/hydroxides and sesquihydroxides/sesquioxides [33]. Compared with CK, the concentrations of SO_4_^2−^ in the topsoil and at the distance of 5 cm and 38 cm from the topsoil with CSF addition increased significantly by 85.2%, 178.4%, and 95.8%, respectively. In rice paddy soil, SO_4_ ^2−^ is likely retained at depth through anion exchange reactions associated with Fe-oxides and Al-oxides [33].

Compared with the CK treatment, adding CSF significantly increased the concentrations of total Fe in the topsoil and the soil 5, 10, 20, and 30 cm away from the topsoil by 384.0%, 544.4%, 358.6%, 430.8%, and 279.9%, respectively, on day 15 (Table 5). The concentrations of total Fe in the soil layer 5 cm away from the topsoil had the highest Fe content with CSF addition. Moreover, the total Fe concentrations decreased with the distance from the topsoil (5 to 30cm). However, there was no significant change in total Fe concentrations between the different soil layers after 100 days of incubation in the CSF treatment. The concentrations of total Fe in the topsoil 5, 10, 30, and 38 cm and away from the topsoil significantly increased by 178.1%, 105.0%, 295.1%, and 159.0%, respectively, after 100 days of the CSF treatment. The Fe contents varied with depth and over time, possibly due to the reduction and dissolution of Fe minerals in the soil and to leaching.

### 3.3. Effect of CSF on Bioavailable Cd, Pb, and As Concentrations

**Figure 2:**
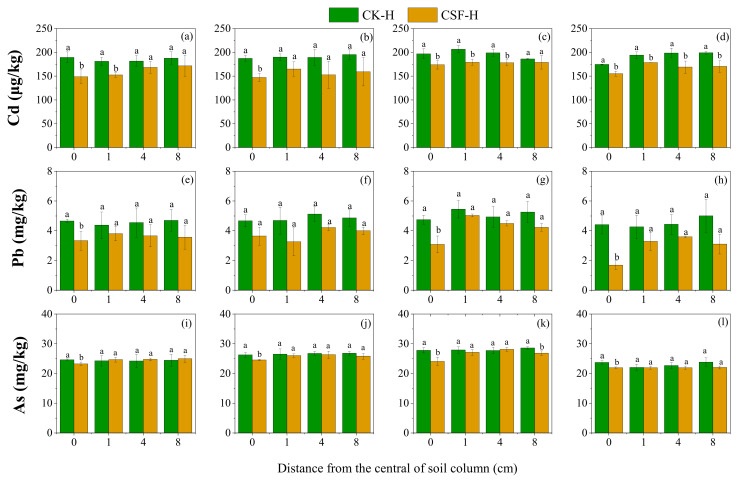
The effect of horizontal migration of CSF on the concentrations of bioavailable Cd, Pb, and As in the surrounding soil, the lowercase letters indicate a significant difference between CK and CSF treatments for the same distance. (**a**,**e**,**i**): Day 15 of incubation; (**b**,**f**,**j**): Day 30 of incubation; (**c**,**g**,**k**): Day 60 of incubation; (**d**,**h**,**l**): Day 100 of incubation.

**Figure 3:**
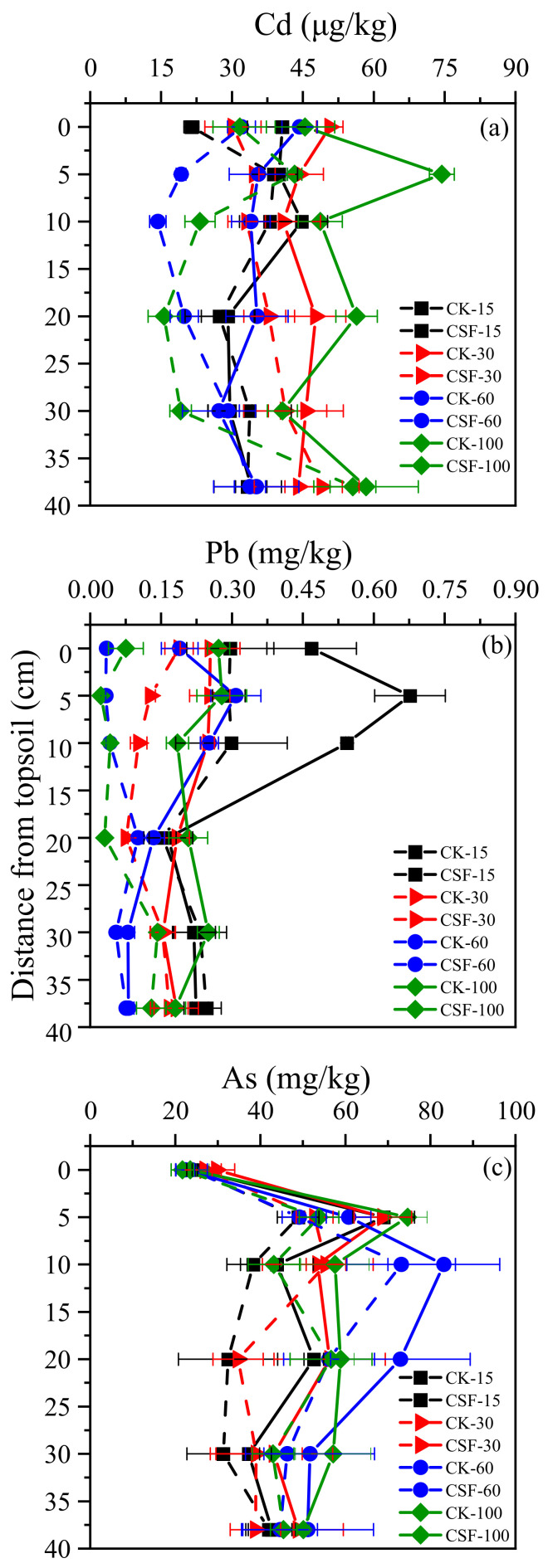
The effect of vertical migration of CSF on the concentrations of bioavailable Cd, Pb, and As in the surrounding soil. (**a**): The concentrations of bioavailableCd in the vertical soil column; (**b**): The concentrations of bioavailable Pb in the vertical soil column; (**c**): The concentrations of bioavailable As in the vertical soil column.

**Figure 4:**
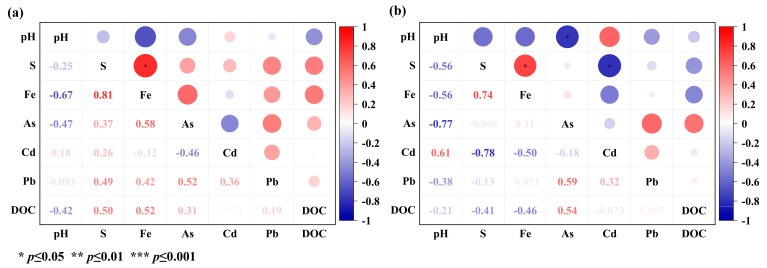
Correlation between physical and chemical properties of soil and bioavailability of Cd, Pb, and As in the horizontal soil column. (**a**): CK; (**b**): CSF.

The addition of CSF in the soil column center or surface of the soil column affected the bioavailability of Cd, Pb, and As in the surrounding space soil (Figure 2 and Figure 3). In the horizontal soil column, CSF treatment decreased the bioavailable Cd concentrations in the soil column center and soil within 1 cm around the soil column center. Although adding CSF had no significant effect on decreasing Cd bioavailability in all the soils except for the soil column center on day 30, the CSF effective range gradually expanded with incubation time (Figure 2a–d). On day 60, the bioavailable Cd concentrations in the CSF treatment at 0 cm, 1 cm, and 4 cm were lower than that at the corresponding distance of the CK, which was significantly decreased by 11.8%, 13.3%, and 10.5%, respectively. Furthermore, on day 100, adding CSF in the soil column center decreased bioavailable Cd concentrations at 8 cm from the central column. The bioavailable Cd concentrations were significantly decreased by 11.1%, 8.1%, 14.9% and 14.3% at 0, 1, 4, and 8 cm away from the soil column center, respectively. When taken together, these results indicate that CSF’s immobilization range for Cd increased over time. The Cd concentration significantly correlated with the SO_4_^2−^ contents (Figure 4b). This correlation may be due to the increase in sulfur, leading to Cd precipitation as CdS [34], resulting in a decrease in bioavailable Cd. The effect of CSF on reducing the bioavailability of Pb and As differed from that on Cd. As shown in Figure 2e–l, compared with CK, within 100 days of incubation, the bioavailable Pb concentrations in the soil supplemented with CSF only significantly decreased at the soil column center. The stabilization efficiency of CSF on Pb in the soil column center increased with incubation time, which was 28.5%, 22.0%, 34.9%, and 61.6% on days 15, 30, 60, and 100, respectively. Adding CSF to the soil column center had no significant effect on the bioavailability of Pb in the surrounding soil. Similarly, adding CSF only decreased the bioavailable As at the central soil column within 100 days of incubation. The concentrations of bioavailable As decreased by 5.6%, 6.7%, 13.4%, and 7.4% on days 15, 30, 60, and 100, respectively. Sulfate (SO_4_^2−^) can be reduced to sulfide (S^2−^) and then immobilize heavy metals, forming a stable sulfide-bound state [35]. The Ksp-CdS (2.6 × 10 ^−29^) is lower than Ksp-PbS (3.4 × 10^−28^) and Ksp-As_2_S_3_ (2.1 × 10^−22^) [36]. Therefore, the combination order of Pb, As, and S^2−^ could be limited by Cd, which could explain the farthest CSF’s stabilization range for Cd.

In the vertical soil column, the concentrations of bioavailable Cd, Pb, and As of the soil column treated with CSF were lower than that of the control treatment (Figure 3). The bioavailable Cd concentration in the CSF treatment was significantly decreased compared to that of the CK in the topsoil on day 15: the CSF’s immobilization depths for Cd increased over time. After 30, 60, and 100 days of incubation, the CSF immobilized Cd at a depth of 5 cm, 10 cm, and 20 cm, respectively. The available Pb in the soil also showed a similar trend to Cd; however, stabilizing was easier. The CSF’s immobilization depths for Pb were 10 cm on day 15; however, it took 60 days for Cd to be at this depth.

**Figure 5:**
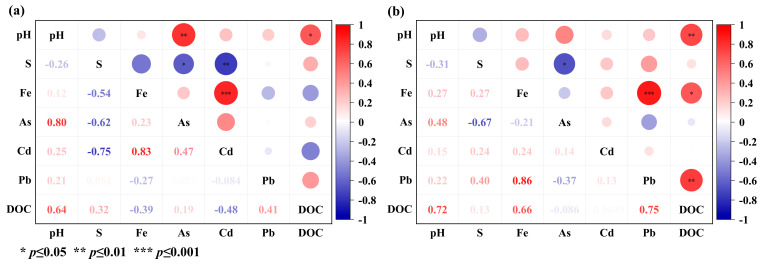
Correlation between physical and chemical properties of soil and bioavailability of Cd, Pb, and As in the vertical soil column. (**a**): CK; (**b**): CSF.

In contrast, Pb’s deepest immobilization depth was 20 cm during the 100 incubation days. The bioavailable Pb concentrations decreased between 36.73% and 91.93%. The concentrations of bioavailable Pb were significantly correlated with total Fe concentration (Figure 5b) with CSF amendment. The greater amount of Pb in the soil than Cd and As would compete with Cd and As to adsorb, making it difficult for Cd and As to combine with CSF. Moreover, the bioavailable As concentrations varied with depths, whereby the concentrations increased and then decreased in all depth layers. The immobilization depths for As were 5 to 10 cm during the 100 incubation days. In addition, regardless of whether CSF was added, the bioavailable As content at 0 cm in the two treatments at the four time points was significantly lower than that at other distances. We speculated that the As content in the topsoil leached into the underlying soil, consequently increasing the bioavailable As contents in the underlying soil over time. Furthermore, the oxic condition of the topsoil may have promoted As precipitation and adsorption of soil mineral oxides [37].

## 4. Conclusions

This study evaluated the spatial and temporal variations of Cd, Pb, and As bioavailability in paddy soils regulated by CSF. The results showed that CSF decreased the bioavailable Cd, Pb, and As concentrations in the soil column center. The immobilization range and depth for Cd, Pb, and As were different, and the immobilization effect of CSF for Cd was the best. The physical and chemical properties of soil, such as soil particle size fraction and perviousness, may influence the migration of the CSF, which influence the immobilization range and depth for heavy metal. Therefore, the immobilization range and depth for heavy metal by CSF in different soils must be further investigated. This study provides important insights into the application frequency and spacing distance when using CSF to immobilize heavy metals.

## Figures and Tables

**Table 1:** Basic physical and chemical characteristics of the tested soil and CSF.

	pH	Organic Matter (g/kg)	Soil Grain Diameter (%)			Cd (mg/kg)	Pb (mg/kg)	As (mg/kg)
			Sand	Clay	Silt			
Soil	6.48	43.33	10.70	76.40	12.90	0.54	416.58	94.20
Calcium sulfate	8.81	-	-	-	-	nd	nd	5.84
Ferric oxide	3.29	-	-	-	-	nd	1.27	nd

nd: Not detected; (-) = Not measure data.

**Table 2:** The change of pH in different horizontal soil layers with CSF addition.

Time	Treatment	Distance from the Center of the Soil Column			
		0 cm	1 cm	4 cm	8 cm
15 d	CK-H	5.56 ± 0.30 Aa	5.27 ± 0.30 Aa	5.27 ± 0.30 Aa	5.28 ± 0.28 Aa
	CSF-H	4.88 ± 0.01 Ab	5.00 ± 0.11 Aa	5.01 ± 0.12 Aa	5.00 ± 0.10 Aa
100 d	CK-H	5.75 ± 0.23 Aa	5.58 ± 0.23 Aa	5.58 ± 0.32 Aa	5.57 ± 0.25 Aa
	CSF-H	5.16 ± 0.14 Ab	5.24 ± 0.19 Aa	5.27 ± 0.24 Aa	5.23 ± 0.26 Aa

Note: The capital letters indicate a significant (*p* < 0.05) difference between the different distances for the same treatments, and the lowercase letters indicate a significant difference between CK and CSF treatments for the same distance.

**Table 3:** The pH changes in the different vertical soil layers with CSF addition.

Time	Treatment	Distance from the Topsoil					
		0 cm	5 cm	10 cm	20 cm	30 cm	38 cm
15 d	CK-V	5.52 ± 0.10 Ba	6.90 ± 0.08 Aa	6.83 ± 0.29 Aa	6.21 ± 0.94 Aa	6.10 ± 0.98 Aa	6.55 ± 1.19 Aa
	CSF-V	5.46 ± 0.03 Ca	6.96 ± 0.09 ABa	6.70 ± 0.10 Ba	7.12 ± 0.07 Aa	7.02 ± 0.16 Aa	7.16 ± 0.30 Aa
100 d	CK-V	5.24 ± 0.09 Ba	6.66 ± 0.21 Aa	6.46 ± 0.04 Aa	6.68 ± 0.16 Aa	6.27 ± 0.59 Aa	6.34 ± 0.89 Aa
	CSF-V	5.25 ± 0.03 Ca	6.24 ± 0.08 Bb	6.31 ± 0.08 Ba	6.68 ± 0.12 Aa	6.82 ± 0.20 Aa	6.73 ± 0.08 Aa

Note: The capital letters indicate a significant (*p* < 0.05) difference between the different distances for the same treatments, and the lowercase letters indicate a significant difference between CK and CSF treatments for the same distance.

**Table 4:** The concentrations of SO_4_^2−^ and total Fe in different horizontal soil layers with CSF addition.

Element	Time	Treatment	Distance from the Center of the Soil Column			
			0 cm	1 cm	4 cm	8 cm
SO_4_^2−^ (mg/L)	15 d	CK-H	11.93 ± 2.53 Ab	9.29 ± 1.29 Ab	12.48 ± 3.97 Aa	11.84 ± 3.44 Aa
		CSF-H	86.86 ± 2.95 Aa	39.60 ± 6.83 Ba	16.96 ± 4.47 Ca	12.01 ± 2.44 Ca
	100 d	CK-H	9.37 ± 2.73 Ab	7.87 ± 1.81 Ab	13.10 ± 2.56 Ab	11.11 ± 1.25 Ab
		CSF-H	36.24 ± 3.06 Aa	26.44 ± 1.42 Ba	20.34 ± 3.27 Ca	20.70 ± 3.29 Ca
Fe (μg/L)	15 d	CK-H	15.87 ± 3.87 Ab	15.57 ± 8.60 Aa	23.63 ± 13.61 Aa	21.40 ± 5.17 Aa
		CSF-H	32.17 ± 6.33 Aa	23.60 ± 1.73 BCa	16.63 ± 0.85 Ca	26.80 ± 4.23 ABa
	100 d	CK-H	13.23 ± 8.04 Aa	9.27 ± 6.11 Ab	17.87 ± 4.76 Aa	16.10 ± 1.91 Aa
		CSF-H	23.80 ± 4.16 Aa	23.93 ± 6.84 Aa	20.07 ± 8.70 Aa	18.37 ± 3.51 Aa

Note: The capital letters indicate a significant (*p* < 0.05) difference between the different distances for the same treatments, and the lowercase letters indicate a significant difference between CK and CSF treatments for the same distance.

**Table 5:** The concentrations of SO_4_^2−^ and total Fe in different vertical soil layers with CSF addition.

Element	Time	Treatment	Distance from the Topsoil					
			0 cm	5 cm	10 cm	20 cm	30 cm	38 cm
SO_4_^2−^ (mg/L)	15 d	CK-V	1.60 ± 0.42 ABb	1.04 ± 0.08 Ba	1.30 ± 0.20 ABa	1.24 ± 0.47 Ba	1.79 ± 0.42 ABa	2.12 ± 0.70 Aa
		CSF-V	2.79 ± 0.56 Aa	0.87 ± 0.16 Ba	1.15 ± 0.14 Ba	1.52 ± 0.06 Ba	2.05 ± 1.1 ABa	2.00 ± 0.86 ABa
	100 d	CK-V	1.08 ± 0.43 ABb	0.37 ± 0.01 Cb	0.59 ± 0.24 Ca	0.70 ± 0.07 BCa	1.25 ± 0.23 Aa	1.19 ± 0.22 Ab
		CSF-V	2.00 ± 0.34 Aa	1.03 ± 0.11 Ba	0.62 ± 0.14 Ca	0.77 ± 0.22 BCa	1.07 ± 0.12 Ba	2.33 ± 0.21 Aa
Fe (μg/L)	15 d	CK-V	7.58 ± 1.72 Ab	7.41 ± 2.82 Ab	7.61 ± 1.43 Ab	6.53 ± 2.33 Ab	6.80 ± 2.36 Ab	7.49 ± 3.09 Aa
		CSF-V	36.69 ± 8.20 ABa	47.75 ± 3.29 Aa	34.90 ± 10.79 ABa	34.66 ± 7.42 ABa	25.83 ± 11.48 Ba	12.36 ± 4.83 Ba
	100 d	CK-V	8.51 ± 2.38 Ab	9.57 ± 2.50 Ab	9.00 ± 3.49 Aa	9.51 ± 4.18 Aa	6.80 ± 2.14 Ab	11.21 ± 2.63 Ab
		CSF-V	23.67 ± 7.4 Aa	19.62 ± 6.58 Aa	17.60 ± 6.45 Aa	17.77 ± 5.86 Aa	26.87 ± 4.93 Aa	29.03 ± 12.43 Aa

Note: The capital letters indicate a significant (*p* < 0.05) difference between the different distances for the same treatments, and the lowercase letters indicate a significant difference between CK and CSF treatments for the same distance.

## Data Availability

Not applicable.
